# Decreased DNA Methylation in the Shati/Nat8l Promoter in Both Patients with Schizophrenia and a Methamphetamine-Induced Murine Model of Schizophrenia-Like Phenotype

**DOI:** 10.1371/journal.pone.0157959

**Published:** 2016-06-27

**Authors:** Kyosuke Uno, Yuu Kikuchi, Mina Iwata, Takashi Uehara, Tadasu Matsuoka, Tomiki Sumiyoshi, Yoshinori Okamoto, Hideto Jinno, Tatsuyuki Takada, Yoko Furukawa-Hibi, Toshitaka Nabeshima, Yoshiaki Miyamoto, Atsumi Nitta

**Affiliations:** 1 Department of Pharmaceutical Therapy and Neuropharmacology, Faculty of Pharmaceutical Sciences, University of Toyama, Toyama, Japan; 2 Department of Neuropsychiatry, Kanazawa Medical University, Kahoku, Japan; 3 Department of Neuropsychiatry, University of Toyama Graduate School of Medicine and Pharmaceutical Sciences, Toyama, Japan; 4 Medical Corporation Syoufukai, Matsuoka Hospital, Oyabe, Japan; 5 Department of Clinical Epidemiology, Translational Medical Center, National Center of Neurology and Psychiatry, Tokyo, Japan; 6 Faculty of Pharmacy, Meijo University, Nagoya, Japan; 7 Department of Pharmaceutical Sciences, Ritsumeikan University, Kusatsu, Japan; 8 Department of Neuropsychopharmacology and Hospital Pharmacy, Nagoya University Graduate School of Medicine, Nagoya, Japan; 9 Advanced Diagnostic System Research Laboratory, Fujita Health University, Graduate School of Health Sciences & Aino University, Toyoake & Ibaragi, Japan; Chiba University Center for Forensic Mental Health, JAPAN

## Abstract

The number of patients with schizophrenia has increased over the past decade. Previously, many studies have been performed to establish its diagnostic criteria, prophylactic methods, and effective therapies. In this study, we analyzed whether the ratios of DNA methylation in CpG islands of the *Shati/Nat8l* is decreased in model mice of schizophrenia-like phenotype using genomic DNA collected from brain regions and peripheral blood, since the mouse model of schizophrenia-like phenotype, mice treated repeatedly with methamphetamine showed increase of Shati/Nat8l mRNA expression in our previous experiment. The ratios of *Shati/Nat8l* CpG island methylation were significantly decreased in both the nucleus accumbens and the peripheral blood of model mice compared with those of control mice. We also investigated *Shati/Nat8l* methylation in the blood of patients with schizophrenia. We found that *Shati/Nat8l* CpG island methylation ratios were lower in the patients with schizophrenia than in the healthy controls, which is consistent with our findings in the mice model. To our knowledge, this is the first study to show similar alterations in methylation status of a particular genomic DNA site in both the brain and peripheral blood of mice. Furthermore, the same phenomenon was observed in corresponding human genomic sequences of the DNA extracted from the peripheral blood of patients with schizophrenia. Based on our findings, DNA methylation profiles of the CpG island of *Shati/Nat8l* might be a diagnostic biomarker of schizophrenia.

## Introduction

In recent years, the number of patients diagnosed with schizophrenia has increased in many countries. Previously, many studies have been performed to improve its prophylaxis, diagnosis, and treatment. Long-term medical treatments are required for these patients; however, due to the lack of accurate laboratory tests, early diagnosis of schizophrenia has not been possible. Currently, several psychiatric diseases, including schizophrenia, are diagnosed primarily through a diagnostic interview and/or an imaging analysis. Given that many patients lack subjective symptoms during the early stage of schizophrenia, it is uncommon for them to be referred to a psychiatric specialist, which is necessary for an early diagnosis. In psychiatric diseases, many changes occur in the brain. For example, it has been reported that *SOX10* [1] and monoamine oxidase A and B [2] were methylated to a greater extent in the postmortem brains of patients with schizophrenia than in healthy controls. These results cannot be directly applied to the diagnosis of psychiatric diseases because obtaining a tissue sample from the brains of living patients is not feasible for the analysis of methylation status.

In this study, we focused on *Shati/Nat8l* as a potential genetic marker associated with psychiatric diseases. The expression of *Shati/Nat8l* was significantly increased in the nucleus accumbens (NAc) of mice treated with methamphetamine (Meth), which disrupts the formation of drug dependence [3–6]. It has been pointed out that a schizophrenia-like symptom has been observed in both mice and humans who have received chronic Meth treatment [7–9]. Therefore, we believe that *Shati/Nat8l* could be associated with psychiatric diseases, which can serve as a diagnostic marker. However, it is too difficult to measure its mRNA or protein levels in the brains of living patients within the traditional clinical setting.

With respect to cancer biology, epigenetic changes, without concomitant changes to genomic sequences, are an important factor for the understanding of mechanisms of cancer onset [10], which may serve as diagnostic markers. Some studies have indicated the possibility of applying epigenetic strategies to psychiatric diseases [11, 12]. One important component of epigenetics is DNA methylation, which is particularly found in the cytosine residues of the CpG (5'-CG-3') arrangement in DNA [13]. Domains that include large amounts of CpG have been termed as CpG Island (CpG Is). CpG Is is usually the upstream sites of the transcription initiation site where methylation takes place [14]. It is generally considered that transcription decreases when there is a higher amount of DNA methylation.

In this study, we focused on DNA methylation of *Shati/Nat8l* and analyzed the methylation ratio in CpG Is upstream of the *Shati/Nat8l* transcription start site, since the expression of Shati/Nat8l was increased in the NAc of mice treated with Meth [3]

## Materials and Methods

### Animals and Meth treatment

All experiments were performed on 8-week old male C57BL/6J inbred mice weighing about 20–25 g that were acquired from Nihon SLC Inc., Japan (Shizuoka, Japan). Mice were kept in a temperature and humidity-controlled environment with 12 h light/dark cycle (lights on at 8:00) and had access to food and water *ad libitum*. All mice received repeated treatment of methamphetamine were quickly decapitated by animal guillotine without feeling any suffering, since the fresh brain tissues were needed for the isolation of genomic DNA. This procedure were done without anesthesia to avoid the effect of anesthetic drugs. All procedures followed the National Institute of Health Guideline for the Care and Use of Laboratory Animals and were approved by the committee for Animal Experiments of the University of Toyama (*Permit Number A2012-PHA31)*.

Mice were administered with saline or Meth hydrochloride (Dainippon Sumitomo Pharmaceutical Co, Osaka, Japan) at a dosage of 1 mg/kg for 6 days and decapitated 2 h after the last administration of Meth for bisulfate sequencing and quantitative real-time RT-PCR.

### Quantitative RT-PCR

Quantitative RT-PCR was performed according to the previous reports [5]. Briefly, total RNA extraction was performed using ISOGEN-LS (NIPPON GENE, Tokyo, Japan). Total RNA from the brain tissues of mice were transcribed into cDNA using the Prime Script RT reagent kit (Takara, Shiga, Japan) according to the manufacturer’s recommendation. Real-time PCR was performed with SYBR Green-based reagents (Thunder Bird SYBR qPCR Mix, Toyobo, Tokyo, Japan), using a Takara Dice Real Time System (Takara). The reaction was performed as follows: 1 cycle at 95°C for 5 min; 45 cycles at 95°C for 20 s, 60°C for 20 s, and 72°C for 20 s. Primer sequences used for PCR were as follows: 5′-GTGATTCTGGCCTACCTGGA-3′ and 5′-CCACTGTGTTGTCCTCCTCA-3′ as mouse *Shati/Nat8l* primers (up and down), 5′-ACCCTGAAGTGCTCGACATC-3′ and 5′-AGGAAGGCCTTGACCTTTTC-3′ as mouse 36B4 primers (up and down).

### Isolation of Genomic DNA

Genomic DNA from the brain was isolated using Extract-N-Amp Tissue PCR Kit (Sigma-Aldrich, St. Louis, MO) according to the manufacturer’s instructions. Genomic DNA from cardiac blood was isolated using Gentra Puregene Blood Kit (QIAGEN, Hilden, Germany) according to the manufacturer’s instructions.

### Bisulfite Conversion

DNA (1 μg) was treated with sodium bisulfite using an EZ DNA Methylation Kit (Zymo Research, Orange, CA) according to the manufacturer’s instructions. Briefly, DNA, bisulfite mix, and DNA protect buffer were mixed together. The bisulfite conversion thermal cycling conditions were as follows: 99°C for 5 min, 60°C for 25 min, 99°C for 5 min, 60°C for 85 min, 99°C for 5 min, and 60°C for 175 min. Finally, the bisulfite-converted DNA was purified on a spin column and eluted with 20 μL of elution buffer.

### Amplification of PCR Product

PCR primers were designed using Methyl Primer Express Software v1.0 (Applied Biosystems Inc., Foster City, CA https://products.appliedbiosystems.com/ab/en/US/adirect/ab?cmd=catNavigate2&catID=602121&tab=Overvie). PCR primer sets included sense (5′-GGGATTTAGGAGAAGTTAATGTG-3′) and antisense (5′-ACATATCCACTTTCCTAAATCACA-3′) primers. Thermal cycling conditions were as follows: 1 cycle at 94°C for 5 min, 35 cycles at 98°C for 30s, 57°C for 1 min, and 72°C for 2 min, 1 cycle at 72°C for 10 min.

### Ligation, Transformation, and Sequence

PCR products were inserted into a pGEM-T Easy Vector System I (Promega, Madison, WI) by Ligation high ver. 2 (Toyobo, Osaka, Japan) according to the manufacturer’s instructions. Colonies were picked and cultured overnight in LB medium containing 50 μg/mL ampicillin. We isolated the plasmid DNA using a boiling mini-prep method, and the sequence was analyzed using both the T7 primer forward and reverse sequencing primers.

### Sequence Analysis

DNA sequencing was performed with a BigDye terminator v1.1 cycle sequencing kit (Applied Biosystems Inc.) according to the manufacturer’s instructions. The T7 sequencing primer (Invitrogen, Carlsbad, CA, USA) and the sense primer 5′-TAATACGACTCACTATAGGG-3′ were used. The DNA-sequencing reactions in 96 well plates were run on a 3130 genetic analyzer (Applied Biosystems Inc.), and the resulting sequences were analyzed with sequencing analysis software (Applied Biosystems Inc.).

### The Human Genomic Methylation Experiments

Seven Japanese schizophrenia male patients and seven healthy male volunteers were recruited from the outpatient unit of The University of Toyama Hospital. The clinical study protocol was conducted in accordance with the Declaration of Helsinki and its Amendments, and was approved by the institutional review board at the University of Toyama (*Permit Number* 24–6). Written informed consent was obtained from all the participants. Genomic DNA were collected from blood. Genomic DNA isolation and bisulfate sequence was performed as mentioned above. PCR primer for cloning into pGEM-T easy was as follows: human *Shati/Nat8l* up primer as 5′-GGAGTTATGTGGGATTTTTAAAGA-3′ and human *Shati/Nat8l* down primer as 5′-ACCTAACCCCCTTCAATTCTAC-3′.

### Statistical Analysis

All data for mice are displayed as the mean ± SEM. Statistical differences between the two groups were determined with a Student’s-*t* test. In human methylation analysis, two groups were determined with an *F* test, followed by Student’s-*t* test.

## Results

### *Shati/Nat8l* mRNA expression in the NAc was increased in model mice of schizophrenia-like phenotype induced by repeated Meth administration

**Fig 1:**
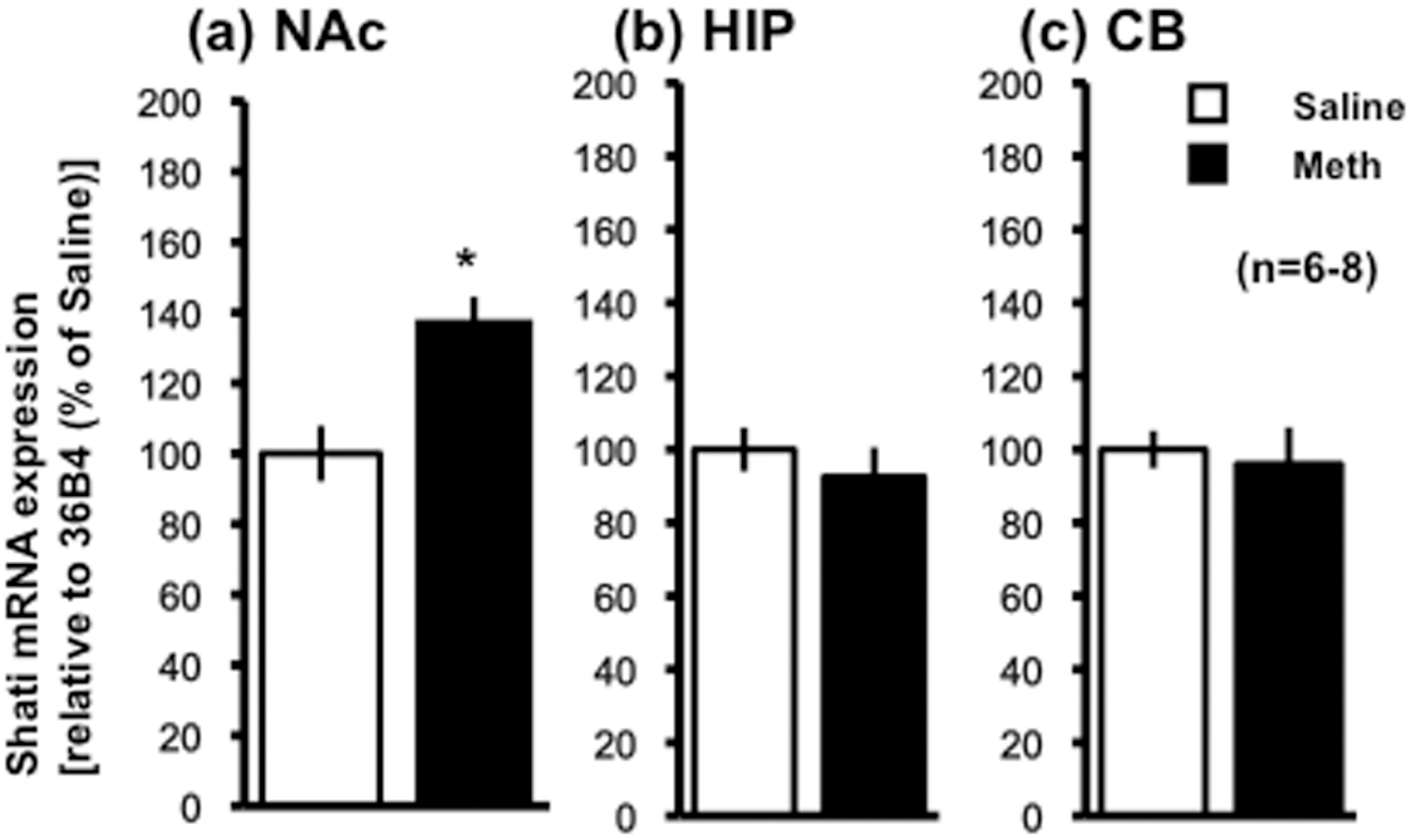
Relative expression of Shati/Nat8l mRNA in mice administered saline or Meth. Real-time RT-PCR was performed to quantify Shati/Nat8l mRNA in mice treated with saline (white bar) or MAP (black bar) from (a) nucleus accumbens (NAc), (b) hippocampus (HIP) and (c) cerebellum (CB). Primers used for each PCR are shown in the text. Data were normalized to 36B4 expression. The fold-change in expression of Shati/Nat8l mRNA was calculated using the ΔΔCT method. Data were plotted as the mean ± SEM. n = 6–8 mice. Significance is set at *p < 0.01.

To confirm our previous results [3], we determined which brain regions show *Shati/Nat8l* mRNA upregulation by repeated administration of Meth to the mice. Shati/Nat8l mRNA expression was increased by the repeated administration of Meth (1 mg/kg for 6 days, s.c.) in the NAc (Fig 1a) 138.12 ± 6.36%, p < 0.05) but not in the hippocampus (HIP) (Fig 1b) (95.99 ± 10.09%) or the cerebellum (CB) (Fig 1c) (92.52 ± 8.08%). These findings were consistent with our previous report [3].

### DNA methylation patterns in CpG Is of the *Shati/Nat8l* promoter region in several mouse brain regions

**Fig 2:**
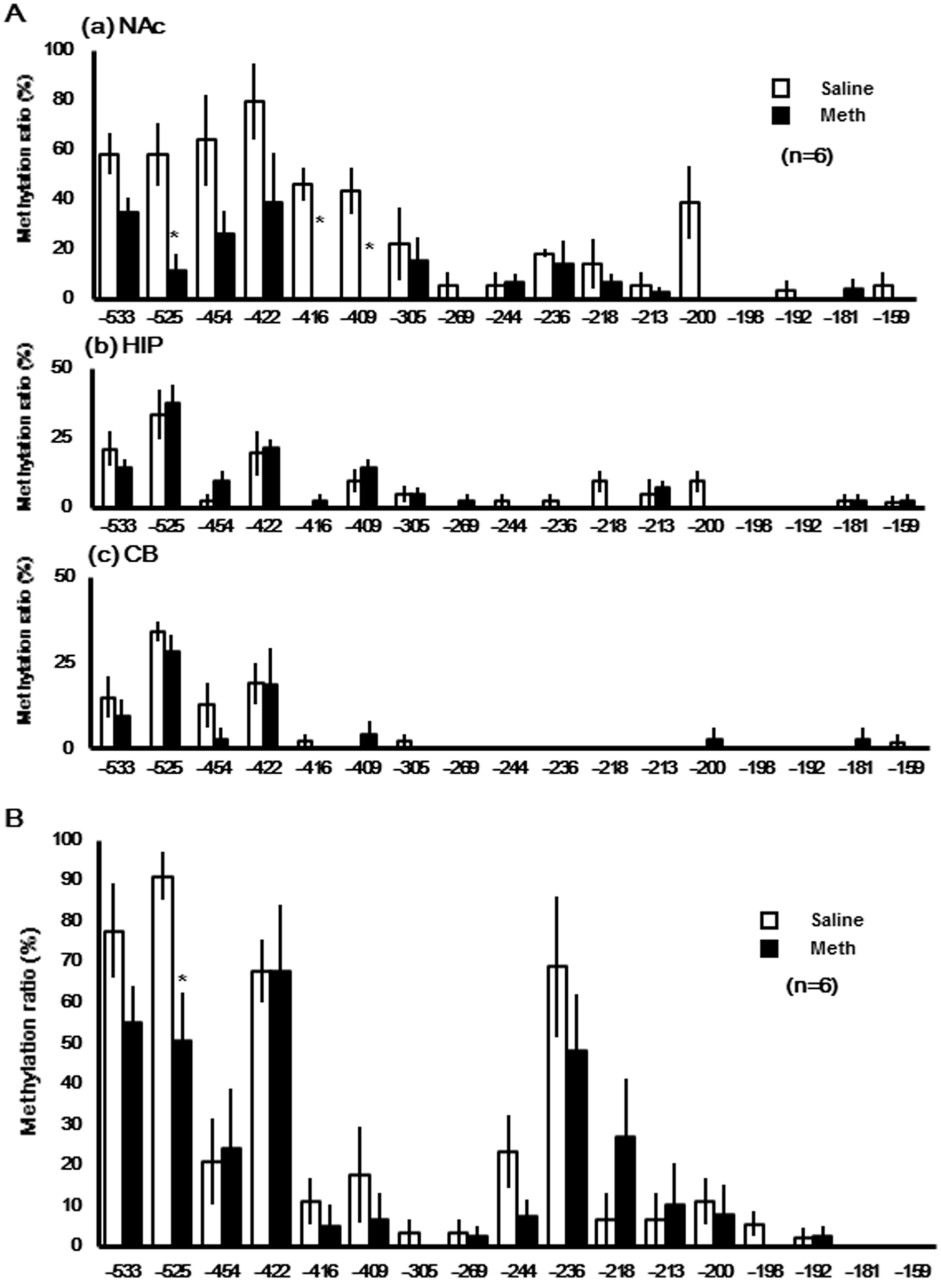
The methylation profiles of *Shati/Nat8l* DNA from mouse brain (A) and cardiac blood (B). The promoter region of *Shati/Nat8* was analyzed in DNA collected from (a) nucleus accumbens (NAc), (b) hippocampus (HIP), (c) cerebellum (CB) and (B) cardiac blood (BLD) of mice. The methylation ratios of each CpG unit and the average of the region are represented. Saline and Meth groups are indicated by white and black bars, respectively. Data are plotted as the mean ± SEM. n = 6 mice. Significance is set at *p < 0.05.

Genomic DNA from three brain regions of mice were isolated and treated with sodium bisulfite, to determine the effect of Meth on DNA methylation in the *Shati/Nat8l*. The resulting DNA was used for methylation-specific PCR using primers designed to detect the methylation status of *Shati/Nat8l* CpG Is. Primers for the CpG Is of *Shati/Nat8l* were designed using Methyl Primer Express (Applied Biosystems,), with the following requirements: %GC >50, CpG Is length: 300~2000 bp, and observed/expected CpG>0.6. According to these requirements, primers for the CpG Is of *Shati/Nat8l* were placed at −562 and +1342 bp from the transcription start site. Therefore, we attempted to analyze the methylation rate of the promoter region of this area (−562 to −135; 17CpGs). As shown in Fig 2A, the methylation ratio of the DNA extracted from NAc of Meth-treated mice was significantly decreased at −525 bp (saline: 58.33 ± 22.05%, Meth: 11.86 ± 11.55%, p < 0.05), −416 bp (saline: 46.30 ± 11.56%, Meth: 0.00%, p < 0.05), and −409 bp (saline: 43.52 ± 16.28%, Meth: 0.00%, p < 0.05) from the *Shati/Nat8l* transcription start site compared with saline-treated mice. However, no significant changes in the methylation status of these genomic regions were observed in the HIP and CB of Meth-treated mice (Fig 2Ab and 2Ac).

### DNA methylation patterns in CpG Is of the *Shati/Nat8l* promoter region in mouse cardiac blood

Next, genomic DNA from mice cardiac blood was isolated and treated with sodium bisulfite. The DNA was sequenced to determine CpG methylation status in cardiac blood from mice treated with Meth. As shown in Fig 2B, CpG methylation in DNA from cardiac blood was significantly decreased at −525 bp (sSline: 58.33 ± 22.05%, Meth: 11.86 ± 11.55%, p < 0.05) from the *Shati/Nat8l* transcription start site in mice that were repeatedly treated with Meth compared with saline-treated mice. Table 1 indicates a typical methylation pattern for individual colonies that were derived from independent bisulfite-treated and PCR-amplified genomic DNA samples. The left half shows the methylation patterns of the saline group and the right half represents the methylation patterns of the MAP group. Open and closed circles indicate unmethylated, and methylated sites, respectively.

**Table 1:** The methylation profiles of *Shati/Nat8l* DNA of mice blood.

	1	2	3	4	5	6	7	8	9	10	11	12	13	14	15	16	17		1	2	3	4	5	6	7	8	9	10	11	12	13	14	15	16	17
**S1**	**●**	**●**	**●**	**●**	**●**	**●**	**●**	**○**	**○**	**●**	**●**	**●**	**●**	**●**	**○**	**○**	**○**	**M1**	**●**	**●**	**○**	**○**	**○**	**○**	**○**	**○**	**○**	**●**	**○**	**○**	**○**	**○**	**○**	**○**	**○**
	**●**	**●**	**○**	**○**	**○**	**○**	**○**	**○**	**○**	**●**	**●**	**●**	**○**	**○**	**○**	**○**	**○**		**●**	**●**	**●**	**●**	**○**	**○**	**○**	**○**	**○**	**○**	**○**	**○**	**○**	**○**	**○**	**○**	**○**
	**●**	**●**	**●**	**●**	**●**	**●**	**○**	**○**	**○**	**○**	**○**	**○**	**●**	**○**	**○**	**○**	**○**		**●**	**●**	**●**	**●**	**○**	**○**	**○**	**○**	**○**	**○**	**○**	**○**	**○**	**○**	**○**	**○**	**○**
	**●**	**●**	**○**	**●**	**○**	**○**	**○**	**○**	**○**	**●**	**○**	**○**	**○**	**○**	**○**	**○**	**○**		**●**	**●**	**●**	**●**	**○**	**○**	**○**	**○**	**○**	**○**	**○**	**○**	**○**	**○**	**○**	**○**	**○**
	**○**	**●**	**○**	**○**	**○**	**○**	**○**	**○**	**○**	**○**	**○**	**○**	**○**	**○**	**○**	**○**	**○**		**●**	**○**	**○**	**●**	**○**	**○**	**○**	**○**	**○**	**○**	**●**	**●**	**●**	**○**	**○**	**○**	**○**
	**○**	**●**	**○**	**○**	**○**	**○**	**○**	**●**	**○**	**○**	**○**	**○**	**○**	**○**	**○**	**○**	**○**		**●**	**○**	**○**	**●**	**●**	**○**	**○**	**○**	**○**	**○**	**○**	**○**	**○**	**○**	**○**	**○**	**○**
	**●**	**●**	**○**	**●**	**○**	**●**	**○**	**○**	**○**	**○**	**○**	**○**	**○**	**○**	**○**	**○**	**○**		**●**	**○**	**○**	**●**	**○**	**○**	**○**	**○**	**○**	**○**	**●**	**●**	**○**	**○**	**○**	**○**	**○**
	**○**	**●**	**○**	**●**	**○**	**●**	**○**	**○**	**●**	**○**	**○**	**○**	**○**	**○**	**○**	**○**	**○**		**●**	**○**	**○**	**●**	**●**	**○**	**○**	**○**	**○**	**○**	**○**	**○**	**○**	**○**	**○**	**○**	**○**
	**●**	**○**	**○**	**●**	**○**	**○**	**○**	**○**	**○**	**●**	**○**	**○**	**○**	**○**	**○**	**○**	**○**		**○**	**○**	**○**	**○**	**○**	**○**	**○**	**○**	**○**	**○**	**●**	**●**	**●**	**○**	**○**	**○**	**○**
	**○**	**○**	**●**	**●**	**○**	**○**	**○**	**○**	**○**	**○**	**○**	**○**	**○**	**○**	**○**	**○**	**○**		**○**	**○**	**○**	**○**	**○**	**○**	**○**	**○**	**○**	**○**	**○**	**○**	**○**	**○**	**○**	**○**	**○**
																			**○**	**○**	**○**	**○**	**○**	**○**	**○**	**○**	**○**	**●**	**○**	**○**	**○**	**○**	**○**	**○**	**○**
**S2**	**○**	**●**	**○**	**○**	**○**	**●**	**○**	**○**	**○**	**○**	**○**	**○**	**●**	**●**	**●**	**○**	**○**		**○**	**○**	**○**	**○**	**○**	**○**	**○**	**○**	**○**	**○**	**●**	**●**	**●**	**○**	**●**	**○**	**○**
	**●**	**●**	**○**	**○**	**○**	**○**	**○**	**○**	**●**	**●**	**○**	**○**	**○**	**○**	**○**	**○**	**○**		**○**	**○**	**○**	**○**	**○**	**○**	**○**	**●**	**●**	**●**	**○**	**○**	**○**	**○**	**○**	**○**	**○**
	**●**	**●**	**●**	**●**	**●**	**●**	**○**	**○**	**○**	**○**	**○**	**○**	**●**	**○**	**○**	**○**	**○**																		
	**●**	**●**	**○**	**○**	**○**	**○**	**○**	**○**	**●**	**●**	**○**	**○**	**○**	**○**	**○**	**○**	**○**	**M2**	**●**	**●**	**○**	**●**	**○**	**○**	**○**	**○**	**○**	**●**	**○**	**○**	**○**	**○**	**○**	**○**	**○**
	**●**	**●**	**○**	**○**	**○**	**○**	**○**	**○**	**●**	**●**	**○**	**○**	**○**	**○**	**○**	**○**	**○**		**●**	**●**	**○**	**●**	**○**	**○**	**○**	**○**	**○**	**●**	**○**	**○**	**○**	**○**	**○**	**○**	**○**
	**●**	**●**	**○**	**○**	**○**	**○**	**○**	**○**	**●**	**●**	**○**	**○**	**○**	**○**	**○**	**○**	**○**		**●**	**●**	**○**	**●**	**○**	**○**	**○**	**○**	**○**	**●**	**○**	**○**	**○**	**○**	**○**	**○**	**○**
	**●**	**●**	**○**	**○**	**○**	**○**	**○**	**○**	**●**	**●**	**○**	**○**	**○**	**○**	**○**	**○**	**○**		**○**	**○**	**○**	**○**	**○**	**○**	**○**	**○**	**○**	**○**	**○**	**○**	**○**	**○**	**○**	**○**	**○**
	**●**	**●**	**●**	**●**	**○**	**○**	**○**	**○**	**○**	**●**	**○**	**○**	**○**	**○**	**○**	**○**	**○**		**●**	**●**	**○**	**○**	**○**	**○**	**○**	**○**	**●**	**●**	**○**	**○**	**○**	**○**	**○**	**○**	**○**
	**○**	**●**	**○**	**●**	**○**	**○**	**○**	**○**	**○**	**●**	**○**	**○**	**○**	**○**	**○**	**○**	**○**		**●**	**●**	**○**	**●**	**○**	**○**	**○**	**○**	**○**	**●**	**○**	**○**	**○**	**○**	**○**	**○**	**○**
	**○**	**○**	**○**	**●**	**○**	**○**	**○**	**○**	**○**	**○**	**○**	**○**	**○**	**○**	**○**	**○**	**○**		**○**	**○**	**○**	**○**	**○**	**○**	**○**	**○**	**○**	**○**	**○**	**○**	**○**	**○**	**○**	**○**	**○**
	**●**	**●**	**○**	**○**	**○**	**○**	**○**	**○**	**●**	**●**	**○**	**○**	**○**	**○**	**○**	**○**	**○**																		
	**●**	**●**	**●**	**●**	**○**	**○**	**○**	**○**	**○**	**●**	**○**	**○**	**○**	**○**	**○**	**○**	**○**	**M3**	**○**	**○**	**●**	**●**	**○**	**○**	**○**	**○**	**○**	**●**	**●**	**○**	**○**	**○**	**○**	**○**	**○**
	**●**	**●**	**●**	**●**	**●**	**○**	**○**	**○**	**○**	**○**	**○**	**○**	**○**	**○**	**○**	**○**	**○**		**○**	**○**	**●**	**●**	**○**	**○**	**○**	**○**	**○**	**●**	**●**	**○**	**○**	**○**	**○**	**○**	**○**
	**○**	**●**	**○**	**●**	**○**	**○**	**○**	**○**	**○**	**●**	**○**	**○**	**○**	**○**	**○**	**○**	**○**		**○**	**○**	**●**	**●**	**○**	**○**	**○**	**○**	**○**	**●**	**●**	**○**	**○**	**○**	**○**	**○**	**○**
	**●**	**●**	**●**	**●**	**○**	**○**	**○**	**○**	**○**	**○**	**○**	**○**	**○**	**○**	**○**	**○**	**○**		**○**	**○**	**●**	**●**	**○**	**○**	**○**	**○**	**○**	**●**	**●**	**○**	**○**	**○**	**○**	**○**	**○**
																			**●**	**●**	**○**	**●**	**○**	**○**	**○**	**○**	**○**	**○**	**○**	**○**	**○**	**○**	**○**	**○**	**○**
**S3**	**●**	**●**	**○**	**○**	**○**	**○**	**○**	**○**	**●**	**●**	**○**	**○**	**○**	**○**	**○**	**○**	**○**		**●**	**●**	**○**	**●**	**○**	**●**	**○**	**○**	**○**	**○**	**○**	**○**	**○**	**○**	**○**	**○**	**○**
	**●**	**●**	**○**	**●**	**○**	**○**	**○**	**○**	**○**	**●**	**○**	**○**	**○**	**○**	**○**	**○**	**○**		**○**	**○**	**●**	**●**	**○**	**○**	**○**	**○**	**○**	**●**	**●**	**○**	**○**	**○**	**○**	**○**	**○**
	**●**	**●**	**○**	**●**	**○**	**○**	**○**	**○**	**○**	**●**	**○**	**○**	**○**	**○**	**○**	**○**	**○**		**○**	**●**	**○**	**●**	**○**	**○**	**○**	**○**	**○**	**○**	**○**	**○**	**○**	**○**	**○**	**○**	**○**
	**●**	**●**	**○**	**●**	**○**	**○**	**○**	**○**	**○**	**●**	**○**	**○**	**○**	**○**	**○**	**○**	**○**		**●**	**●**	**○**	**●**	**○**	**○**	**○**	**○**	**○**	**○**	**○**	**○**	**○**	**○**	**○**	**○**	**○**
	**●**	**●**	**○**	**●**	**○**	**○**	**○**	**○**	**○**	**●**	**○**	**○**	**○**	**○**	**○**	**○**	**○**		**●**	**●**	**○**	**●**	**○**	**●**	**○**	**○**	**○**	**○**	**○**	**○**	**○**	**○**	**○**	**○**	**○**

The methylation pattern is shown for individual colonies that were derived from independent bisulfite-treated and PCR-amplified genomic DNA samples. The left half shows methylation pattern of saline group, and the right half presents one of Meth group. Open circles indicate that the site is unmethylated, and closed circles indicate that a site is methylated. The above numbers are corresponding as below. Vertical axis indicate picked up one colony. The target region consists of 17 CpGs from -533 to -159 nucleotides (CpG1 = -533, CpG2 = -525, CpG3 = -454, CpG4 = -422, CpG5 = -416, CpG6 = -409, CpG7 = -305, CpG8 = -269, CpG9 = -244, CpG10 = -236, CpG11 = -218, CpG12 = -213, CpG13 = -200, CpG14 = -198, CpG15 = -192, CpG16 = -181, CpG17 = -159) upstream from the transcription start site. These were numbered 1–17 in the 5' to 3' direction.

### DNA methylation patterns in CpG Is of the *SHATI/NAT8L* promoter region in human peripheral blood from patients with schizophrenia

Primers for the CpG Is of human *SHATI/NAT8l* were designed using Methyl Primer Express (Applied Biosystems) with the following requirements: %GC >50, CpG Is length: 300~4000 bp, and observed/expected CpG>0.6. According to this requirement, the CpG Is of human *SHATI/NAT8L* was defined as −1726 to +2159 bp from the transcription start site. At *in silico* study, these region include nearly the same transcriptional binding site sequence as in mice -533 to -159 region. Therefore, we attempted to analyze the promoter region of this area (−1714 to −1039 bp; 36CpGs). These sequences are not the same as the mouse alignments; however, they do have the same transcription factor-binding sites as mice. As shown in Table 2, the methylation ratio in peripheral blood was significantly lower at −1532, −1509, −1492, and −1480 bp from the transcription start site in patients with schizophrenia compared with that of healthy controls.

**Table 2:** The methylation profiles of *SHATI/NAT8L* DNA from the human blood.

	Position from the transcription start site								
	-1714	-1700	-1696	-1644	-1636	-1628	-1532	-1509	-1492
Subject									
Healthy	28.43±9.96	23.43±6.78	10.43±3.60	33.00±10.81	24.86±8.41	19.14±6.37	13.43±4.91	7.29±2.32	18.43±5.61
Schizophrenia	12.00±2.91	10.14±3.89	4.85±2.58	16.28±1.800	12.57±4.71	8.00±4.11	3.57±1.40#	1.00±1.00#	6.71±2.18#
	-1482	-1480	-1446	-1404	-1364	-1342	-1310	-1299	-1275
Subject									
Healthy	15.57±7.34	13.57±4.21	9.29±2.06	5.71±2.55	4.57±2.16	0.00±0.00	0.71±0.71	0.57±0.57	0.71±0.71
Schizophrenia	9.71±1.34	4.71±2.25#	5.57±2.64	3.57±1.52	2.00±0.95	2.14±1.01	1.42±0.92	1.57±1.57	2.00±0.95
	-1265	-1261	-1239	-1216	-1211	-1197	-1191	-1146	-1127
Subject									
Healthy	6.00±2.39	4.00±1.79	2.14±1.03	2.14±1.03	3.14±1.72	0.71±0.71	0.00±0.00	5.29±2.53	4.43±2.56
Schizophrenia	3.71±2.36	3.57±2.83	2.00±0.95	2.71±0.97	2.00±0.95	1.28±0.84	0.71±0.71	1.42±0.92	1.42±0.92
	-1120	-1111	-1083	-1078	-1074	-1072	-1065	-1049	-1039
Subject									
Healthy	1.14±1.14	0.71±0.71	2.86±1.03	5.43±2.41	1.43±0.95	2.00±0.95	3.57±1.45	0.00±0.00	0.00±0.00
Schizophrenia	1.42±0.92	0.71±0.71	2.00±0.95	2.85±1.48	1.42±0.92	2.71±0.97	3.42±2.82	0.00±0.00	0.00±0.00

Data were plotted as the mean ± SEM. N = 7 subjects. Significance is set at #p < 0.01.

## Discussion

We previously reported that the expression of Shati/Nat8l mRNA was increased in the prefrontal cortex, striatum, and NAc of mice treated with repeated administration of Meth [3]. In addition, we found Shati/Nat8l mRNA in the neuronal cells throughout the specific brain regions of mice using *in situ* hybridization methods [15]. In this study, we found significant decreases in *Shati/Nat8l* promoter CpG Is methylation in DNA extracted from both the NAc and blood of Meth-treated mice. In the HIP and CB, differences in methylation status were not observed in Meth-treated mice. In addition, the methylation ratio in the CB and HIP was lower than that in other brain regions, even in comparison to the control group. As shown in Fig 1, Shati/Nat8l mRNA expression was increased in the NAc but not in the HIP or CB of Meth-treated mice. In mouse brain areas where lower levels of methylation in *Shati/Nat8l* CpG Is was seen, Shati/Nat8l mRNA expression was not affected by the repeated administration of Meth. Thus, additional mechanisms may regulate transcription of *Shati/Nat8l*.

The reactions that drive demethylation of cytosine residues in DNA can include hydroxymethylation [16, 17]. Therefore, in this study we focused on changes in methylation status in the brain tissue and blood.

Significant changes in both the mRNA expression and the methylation ratio of *Shati/Nat8l* in the NAc, which receives dopaminergic neuronal input, were observed. Dopaminergic regulations can be influenced by Meth owing to the increase of monoamines uptake and dopamine production [18]. Everitt and colleagues reported that the changes in the expression levels of dopamine receptors and dopamine transporters in the HIP and CB were different from those in the NAc [19]. It is possible that differences in the regulation of the dopaminergic system among the NAc, HIP and CB contribute to the differences in DNA methylation in these regions.

Most neuronal systems in the NAc consist of GABAergic neurons. With respect to GABAergic neurons, DNA methylation of GAD67 and GABA synthetase is greatly altered by maternal restriction stress in the brains of mice as well as in patients with schizophrenia [20, 21]. Taken together, our results along with previous studies suggest that GABAergic neurons may regulate DNA methylation of *Shati/Nat8l* in response to stress or psychiatric disorder. In this study, the methylation rate of four different CpG Is of *Shati/Nat8l* were significantly decreased in patients with schizophrenia. Further study is necessary to clarify the relationship of these findings with GAD67.

The systems that regulate DNA methylation consist of many molecules, including DNA methyltransferases (DNMTs) [22], TET methylcytosine dioxygenases [23], methylated DNA-binding protein, and MeCP2 [24]. It has been reported that Tet1-KO animals exhibit abnormal hippocampal long-term depression and impaired memory extinction in addition to downregulation of multiple neuronal activity-regulated genes, including Npas4, c-Fos, and Arc [25]. In a human study, Tet1 mRNA and protein levels were upregulated in the brains of postmortem patients with schizophrenia compared with those of healthy controls. DNMTs are enzymes that cause cytosine methylation, and they have been reported to suppress GAD67 mRNA expression via methylation of the promoter region [26]. Furthermore, DNMT expression was upregulated in the peripheral blood of patients with schizophrenia and in the brains of postmortem patients with schizophrenia [20]. MeCP2 can bind to methylated cytosine bases and act not only to suppress gene expression itself, but it can also recruit other suppressive proteins, and suppress the expression of other genes [27, 28]. Administration of psychostimulant drugs modulates many transcription factors, such as DNMT, MeCP2, and HDAC [29, 30], and these transcription factors show decreased DNA binding ability [31]. 5hmC may be especially important in the central nervous system, as it is found in very high levels there [32]. These reports may support the inconsistent results shown in Fig 2A and 2B, that CpG -525 is significantly hypomethylated in both NAc and blood of Meth-treated mice, but CpG -416 and -409 show no DNA methylation-difference in the blood of Meth-treated mice. In our study, *Shati/Nat8l* expression in the NAc was upregulated by Meth exposure and was accompanied by suppressed methylation in CpG Is of *Sh*ati/Nat8l DNA. Future studies should focus on detailing the mechanisms of this process. In future, we also try other psychiatric disease patients such as bipolar, depression and anxiety.

In this study, we report important findings of the effects of repeated administration of psychostimulant drug on DNA methylation of specific gene not only in the brain but also in peripheral blood of mice. These results were correlated with human studies. Blood tests for the methylation of CpG Is of *Shati/Nat8l* might be useful as a method for diagnosing patients with schizophrenia in the future
